# Letters in the forest: global precedence effect disappears for letters but not for non-letters under reading-like conditions

**DOI:** 10.3389/fpsyg.2014.00705

**Published:** 2014-07-17

**Authors:** Thomas Lachmann, Andreas Schmitt, Wouter Braet, Cees van Leeuwen

**Affiliations:** ^1^Center for Cognitive Science, Cognitive and Developmental Psychology Unit, University of KaiserslauternKaiserslautern, Germany; ^2^Experimental Psychology Unit, University of LeuvenLeuven, Belgium

**Keywords:** reading acquisition, global advantage effect, analytic processing, holistic processing, literacy, developmental dyslexia, congruence effect

## Abstract

Normally skilled reading involves special processing strategies for letters, which are habitually funneled into an abstract letter code. On the basis of previous studies we argue that this habit leads to the preferred usage of an analytic strategy for the processing of letters, while non-letters are preferably processed via a holistic strategy. The well-known global precedence effect (GPE) seems to contradict to this assumption, since, with compound, hierarchical figures, including letter items, faster responses are observed to the global than to the local level of the figure, as well as an asymmetric interference effect from global to local level. We argue that with letters these effects depend on presentation conditions; only when they elicit the processing strategies automatized for reading, an analytic strategy for letters in contrast to non-letters is to be expected. We compared the GPE for letters and non-letters in central viewing, with the global stimulus size close to the functional visual field in whole word reading (6.5° of visual angle) and local stimuli close to the critical size for fluent reading of individual letters (0.5° of visual angle). Under these conditions, the GPE remained robust for non-letters. For letters, however, it disappeared: letters showed *no* overall response time advantage for the global level and symmetric congruence effects (local-to-global as well as global-to-local interference). We interpret these results as according to the view that reading is based on resident analytic visual processing strategies for letters.

## INTRODUCTION

The ability to read is built on established visual and auditory skills: in the auditory domain, these skills involve the use of spoken language (Friederici and Lachmann, 2002); in the visual domain, they include the capacity to detect and encode small components and to process them in parallel at the level of objects of a certain complexity. These skills are recruited for, respectively, the processing of letters and words. In being recruited, original skills may become modified (Lachmann, 2002; Dehaene and Cohen, 2007; Dehaene et al., 2010b; Lachmann and van Leeuwen, this issue). For instance, in the auditory domain, the phonological structure of language will gain prominence in the process of learning to read (Serniclaes et al., 2005; Port, 2007; Ventura et al., 2008a; Kolinsky et al., 2012). The question is, whether we can likewise observe modifications of the visual domain that emerge in the process of learning to read.

Normally skilled reading involves special processing strategies for letters; these are habitually funneled into an abstract letter code, i.e., a representation for cross-modal usage, derived from both visual and auditory characteristics (Blomert, 2011; Mittag et al., 2013). Several authors have proposed that in acquiring a normal-level of reading ability, visual processing of letters (more precisely graphemes), is singled out from that of similar non-letter shapes (Lachmann and van Leeuwen, 2004, 2008a, this issue; van Leeuwen and Lachmann, 2004; James et al., 2005; Burgund et al., 2006; Pegado et al., 2011; Duñabeitia et al., 2013; Fernandes et al., 2014). According to our views (Lachmann and van Leeuwen, 2008a, this issue), the special strategy for reading letters involves a preferential association of letters with analytic processing, whereas holistic processing is preferred for non-letter visual shapes.

The latter include pseudo-letters, but also whole written words. In non-lexical serial pattern learning, holistic preference develops as a function of practice (van Leeuwen et al., 1988). For words, this may be the product of reading expertise (Frith, 1985; Ehri, 1998; Wong et al., 2011). As a result, words can be processed via a direct lexical route without grapheme–phoneme conversion (Davelaar et al., 1978; Coltheart, 2007). Because of this we may observe in skilled readers the effects of analytic letter processing mainly in case of letters out of word context or in pseudo- or unfamiliar words, i.e., whenever processes of the single-letter level predominate. Still, this condition constitutes a fundamental phase in the development of skilled reading (Frith, 1985; Ehri, 1998). In expert readers it survives as a fall-back strategy to direct word processing (Coltheart, 2007).

To illustrate the differentiation in letter and non-letter processing: in a *same-different* task, in normally reading children, global symmetry led to faster responses in non-letter dot patterns, whereas symmetry did not affect response speed in letters (Lachmann and van Leeuwen, 2007). Clearly, in skilled reading the holistic property of symmetry has become irrelevant for letters and must be suppressed (e.g., Lachmann, 2002; Dehaene et al., 2010a; Pegado et al., 2011, 2014; Fernandes and Kolinsky, 2013; Borst et al., 2014). Another example is that in flanker studies, congruent flankers facilitate responses to non-letters, whereas they do not in case of letters (Lachmann and van Leeuwen, 2004, 2008a; van Leeuwen and Lachmann, 2004). Holistic processing of non-letters leads to binding of the flankers, whereas such effects are absent due to analytic processing in letters. If such differentiations are a consequence of reading experience, they should be absent in adults who never learned to read (Kolinsky et al., 2011; Lachmann et al., 2012; Fernandes et al., 2014) and moreover, are likely to have developed anomalously in dyslexic children and adults (Lachmann and van Leeuwen, 2007, 2008b; Lachmann et al., 2010; Fernandes et al., 2014; Perea and Panadero, 2014).

### READING AND GLOBAL PRECEDENCE

The preference for analytic letter processing in normal readers is apparently in conflict with some well-known observations in a classical paradigm. This paradigm uses compound, hierarchical figures with both a local and a global structure (Kinchla, 1974; Navon, 1977; see Kimchi, 1992 for a review) that give rise to the well-known global precedence effect (GPE): “*forest before trees,*” to use a common metaphor (Navon, 1977). The global structure in these patterns is a configuration, defined by the spatial relationship between its elements, which all have identical local shapes. The task can involve identification, classification, or discrimination of a target either at the global or local level. Consider, for example, a stimulus described by four triangles arranged in a square pattern. The square pattern consists of the global level (“forest”); the triangles are the local level (“trees”). These compound figures have the advantage that the global and local level can be independently varied: besides a square of triangles, we can have a triangle of squares, a square of squares, and a triangle of triangles (see Navon, 1981a, 2003).

The GPE implies, firstly, that for the global-level targets responses are faster than for the local-level ones (*global advantage* or *global superiority effect*). The second observation pertaining to the GPE is called *asymmetric congruence,* which should be understood as follows: Of the above-mentioned four patterns, the square of squares and the triangle of triangles qualify as congruent and the other two as incongruent. Typically for such patterns, incongruency interferes with the local-level target responses but not with global level ones. This and the global advantage effect together constitute the GPE.

The presence of a GPE leads to the conclusion that the global-level properties are given priority in processing, compared to the local ones (we first see the forest, then the trees). We might want to call this type of processing holistic. Note, however, that the dimensions local–global and analytic-holistic do not necessarily refer to the same construct (Wagemans et al., 2012).

The GPE is mostly observed with compound figures in which the local and global levels both consist of letters (Navon, 1977; Lux et al., 2004; Dulaney and Marks, 2007; see Kimchi, 1992, for an overview). This observation might well be considered in contradiction to our claim that while non-letter shapes are typically processed holistically, letters are processed analytically. We would at least prima facie expect an observer in analytic mode not to give priority in processing to the properties of the global shape – this, even though the present task is not quite the same as reading.

We note, however, that there are reasons to expect analytic processing leading to the disappearance of the GPE under particular circumstances. In spite of its abiding character in the literature, the GPE is modulated by a variety of factors, including (1) *stimulus* factors, such as its absolute and relative size (Kinchla and Wolfe, 1979; Martin, 1979; Lamb and Robertson, 1990; Luna et al., 1995; Amirkhiabani, 1998), number of components (Kimchi and Palmer, 1982; Navon, 1983), and spatial frequency characteristics (LaGasse, 1993; Hübner, 1997); (2) factors involving the *mode of presentation*, such as detectability of the local and global features (Kimchi, 1992), visual hemifield (Amirkhiabani, 1998), eccentricity from the focal point of view (Navon and Norman, 1983; Pomerantz, 1983; Amirkhiabani and Lovegrove, 1996) and positional uncertainty (Lamb and Robertson, 1988); and (3) *individual* factors such as prior set (Kimchi, 1992), order of instruction (Foerster and Tory Higgins, 2005), meaningfulness (Poirel et al., 2006) field-dependence-independence (Poirel et al., 2008a) and the stage of brain-development (Poirel et al., 2011).

With few exceptions (e.g., Poirel et al., 2008b), researchers used either letters or non-letters when testing the effect of various factors on the GPE, rather than systematically comparing letters and non-letters. However, across these studies GP effects appear to differ between letters and non-letters. Whereas the GPE, in particular the global advantage, reliably appears with non-letters (Hughes et al., 1984; Luna et al., 1990; Harrison and Stiles, 2009; Kimchi et al., 2009; Bouvet et al., 2011), with letters it depends on a number of factors. One of these is target placement. The original study by Navon (1977) as well as a number of later studies (e.g., Lux et al., 2004; Volberg and Hübner, 2004; Dulaney and Marks, 2007) involved presentation of the local and global letters away from fixation, in combination with positional uncertainty.

For letter-specific analytic processing, it appears crucial that the targets are presented in central view, without positional uncertainty (Plomp et al., 2010). The reason may be that reading typically occurs in a piecemeal fashion, while the sensory information is close to the locus of fixation (Rayner et al., 1986); parafoveal vision in order to control saccades may be important for reading, but uptake of orthographic information takes place only within central vision (Rayner et al., 1986; Jordan and Martin, 1987; Pollatsek, 1993; Stein et al., 2001; Stein, 2002). If analytic processing of letters is due to reading expertise, we are more likely to find it in conditions where the target is placed centrally in visual field.

Since Navon’s seminal work, several studies have presented compound stimuli in the center of the screen without uncertainty and still obtained a GPE (e.g., Kinchla and Wolfe, 1979; Grice et al., 1983; Poirel et al., 2008b). For letters in these conditions, however, the effects were often found to be unstable, reduced, absent or sometimes even reversed (Kinchla and Wolfe, 1979; Pomerantz, 1983; Lamb and Robertson, 1988, 1990; Amirkhiabani and Lovegrove, 1996; Ahmed and Fockert, 2012; see Kimchi, 1992, 2014, for a review).

Whereas for non-letters effects appear relatively invariant, for letters they crucially depend on the visual angle of the global target. The dependency was consistently observed across a number of studies (Kinchla and Wolfe, 1979; Lamb and Robertson, 1990; Luna et al., 1995; Amirkhiabani and Lovegrove, 1996; see Kimchi, 1992 for a review). Amirkhiabani and Lovegrove (1996) found the GPE to disappear with a visual angle extending a size of between 2.5 and 4.6°. Lamb and Robertson (1990) varied the visual angles of global letters from 1.5 to 12° and found that the global advantage effect with letters is restricted to visual angles smaller than 4.5°. Luna et al. (1995) presented the global letter targets with visual angles of 3, 6, and 12° and found, in agreement with the previous studies, the GPE with letters to be restricted to the small visual angle condition of 3°. With 6° the GPE disappeared and in the 12° condition it reversed. All these results are, by and large, in accordance with the earlier finding by Kinchla and Wolfe (1979) that the GPE with letters reverses from a visual angle of about 6–9° upward. These results make it likely that central presentation of global stimuli between 5 and 6° approximately in size leads to a differentiation between letters and non-letters in their GPE effect. However, since for this type of conditions, no comparison between letters and non-letters has so far been made, this conclusion would be based on indirect evidence.

The few studies that did compare letters and non-letters used either peripheral presentation (Dulaney and Marks, 2007) or, if they used central presentation, did not vary the material systematically (Peresotti et al., 1991 only varied material at the global level) and if they did, they used rather large visual angles for the global level (Poirel et al., 2008b; Beaucousin et al., 2011). In the present study we compared in one experiment letters and non-letters, using central placement and a scale of around 5–6° of visual angle for which we may expect the GPE to disappear for letters but to remain for non-letters.

We predict this discrepancy based on the assumption that analytic processing of letters is associated with reading and thus analytic processing most likely will occur with stimulus dimensions, appropriate for fluent reading. This is because for these conditions the reading specific visual processing strategy is automatized (Lachmann and van Leeuwen, this issue). The crucial 5–6° of visual angle may be related to the fact that the word-level information needs to be captured within the *functional visual field*. This is a restricted area of approximately 5–10° of visual angle around the fixation point. Within this field we can perceive an object and its component parts (Sanders, 1970). This means that the local (letter) and global level can be processed in parallel. The size of the functional visual field depends dynamically on the context and varies with factors such as stimulus complexity, crowding, contrast, and attentional demands (Motter and Simioni, 2008). Under conditions typical of reading, with relatively uniform and densely crowded stimuli, the field is relatively small (Legge et al., 2007). On the other hand, the global level stimulus is not surrounded by any flankers. On balance, this means that the size of a global level of 6–7° of visual angle approximately matches the functional visual field. Therefore, we used this size of the visual angle for the global stimulus in the present study.

As for the size of the local level, there is a *critical threshold* for fluent reading in central vision, which is approximately 0.2/0.3° of visual angle (Legge et al., 1985; Jordan and Martin, 1987). We chose local stimuli in the present study to be of the size of 0.5°. Whereas reading becomes less fluent with still larger stimuli, the chosen size of the local stimuli is still quite appropriate with reading. We propose that under the joint constraints of the *critical threshold* and the* functional visual field*, effects of analytic processing in letters are most likely to be found in centrally placed compound letters, and thus contrast with a GPE for non-letters.

## EXPERIMENT

### PARTICIPANTS

Thirty-seven participants (16 female), all students from the University of Kaiserslautern, Germany (mean age: 26 years; SD: 3), took part in this study. All participants were native speakers of German, had normal hearing and normal or corrected-to-normal vision, and were not diagnosed as having any reading disorder. The study was approved by the ethical committee of the Faculty of Social Sciences of the University of Kaiserslautern. Participants gave written informed consent prior to performing the task, and were paid for their participation.

### MATERIAL

**FIGURE 1:**
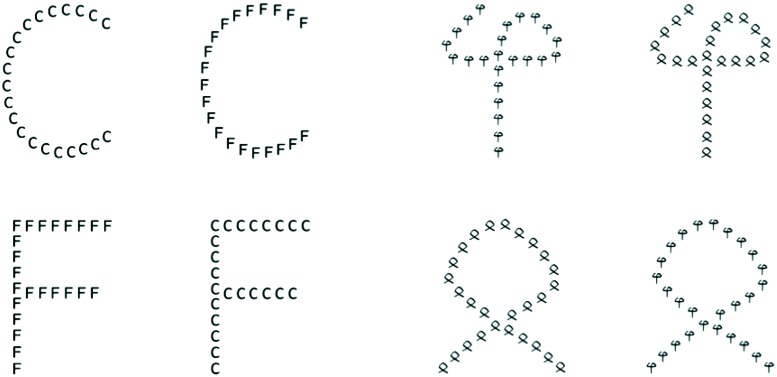
**Illustration of the hierarchical stimuli used in the experiment, left side: letters, right side non-letters.** Leftmost column: congruent letter stimuli, of which the local and the global level consists of the same letters, C or F, respectively. Second column: incongruent letter stimuli, i.e., the global-level letters differ from the local level ones. Third column: congruent non-letter stimuli; right most column: incongruent non-letter stimuli.

Compound, hierarchical (Navon, 1977) letter and non-letter stimuli were used, as illustrated in **Figure 1**. Mixed stimuli (e.g., local letters with global non-letters or vice versa) were not used; the stimuli were either entirely composed of letters (C or F) or of non-letter shapes (the two in **Figure 1**), in all possible hierarchical combinations, which accordingly could be congruent (as far as letters are concerned: a C of Cs or an F of Fs) or incongruent (a C of Fs or an F of Cs, and analogously for non-letters). Stimuli were presented using E-prime 2.0 (Psychology Software Tools, Pittsburg, USA), controlled by a laptop computer running Microsoft Windows XP in a test cubicle with sound attenuation and controlled lighting. The stimuli were presented in black (0.4 cd/m^2^) against a white background (28.9 cd/m^2^), the global stimulus with a visual angle of approximately 6.5° in height and 5.5° in width, the local stimuli with a visual angle of approximately 0.5°.

### DESIGN AND PROCEDURE

Participants performed a two-alternative-forced-choice identification task on the compound, hierarchical letter or non-letter characters. The experimental session consisted of four blocks of 100 trials each. In two blocks, one with letters, and one with non-letters, participants were asked to respond to the identity of the local elements and to ignore the global shape, while in the remaining two blocks, again, one with letters, and one with non-letters, they were instructed to identify the global stimulus while ignoring the local elements. Participants responded by pressing the left key of the embedded laptop mouse with the left index finger or with the right key using their right index finger to the response alternatives, which depended on the specific instructions for a block (e.g., level = global: “F” = left key, “C” = right key). Each block contained 50 congruent trials, i.e., when the identity of the local and the global elements were matched (e.g., global “F” target consisted of local “F” elements) and 50 incongruent trials, i.e., when the identity of the local and global elements were not matched (e.g., local “F” targets formed a global “C” letter shape; see **Figure 1**). Each block started with an instruction screen on which all four possible target figures and the correct responses were shown, respectively.

Each trial started with a fixation cross displayed for 250 ms, followed by a blank screen (250 ms), after which, at the location were the fixation cross had been presented, the compound, hierarchical figure was displayed centrally and without positional uncertainty, until the participant responded (or for 2000 s in case no response was given), followed by another blank screen for 250 ms. Eight practice trials were performed prior each block for which a visual feedback for correct and incorrect responses was given, displayed for 500 ms. All conditions were randomized for each participant or counterbalanced between participants.

## RESULTS

**FIGURE 2:**
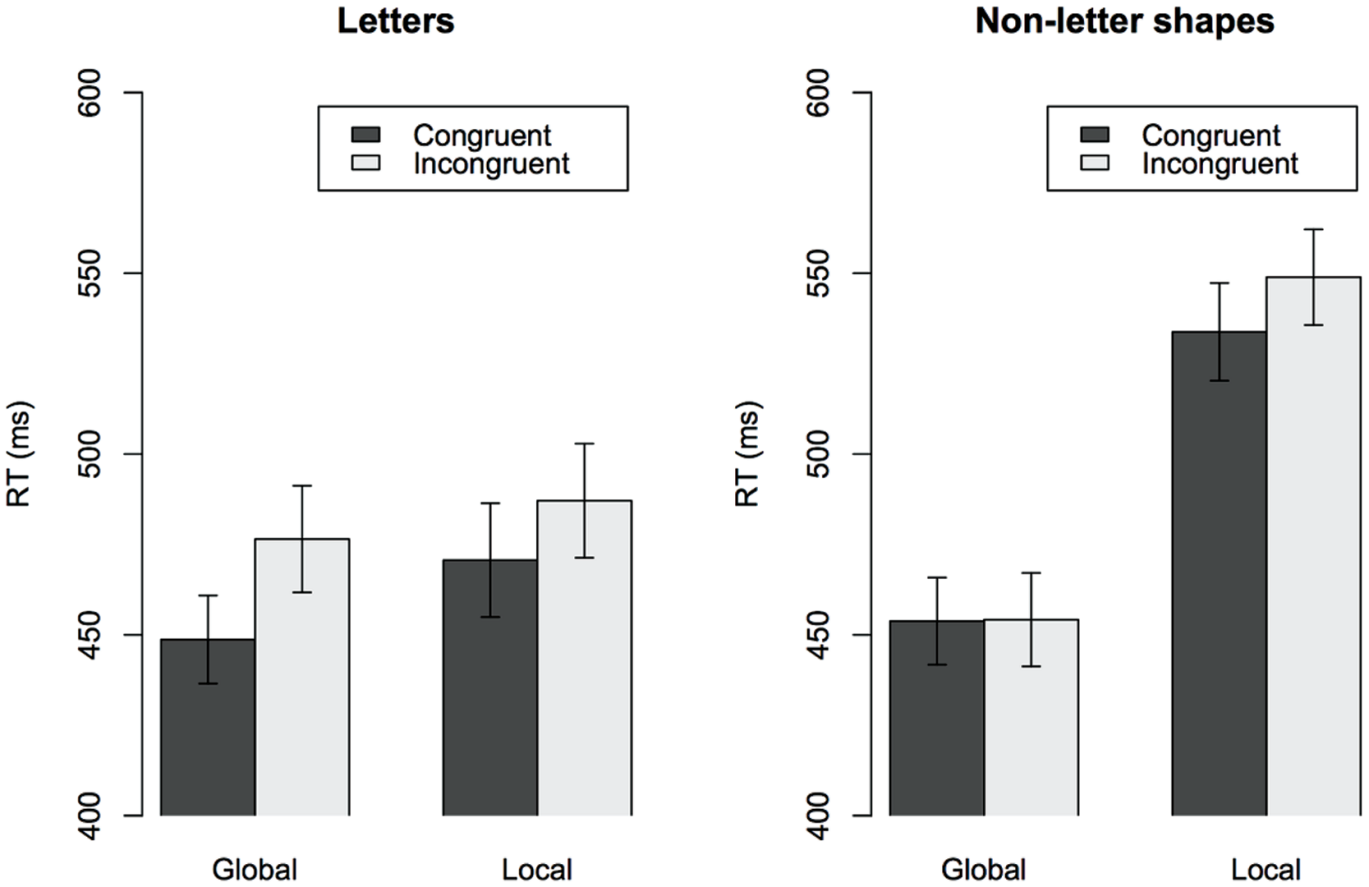
**Mean RT in ms for letter stimuli (left side) and non-letter stimuli (right side) for congruent and incongruent trials in the local and global trial blocks**.

Reaction times (RT) of correct responses within a range of 200-2000 ms and Error Rates were analyzed. No outliers needed to be excluded. There was no evidence for a speed-accuracy trade off, *r*(35) = 0.3 ns. Therefore, in the following sections we will report RT analyses only. The RT data were analyzed by means of repeated-measures Analysis of Variance (ANOVA) with the following factors: Material (letters or non-letter shapes), Level (global or local target), and Congruency (congruent or incongruent); preliminary analyses showed no differences between individual letters or shapes within the letter or non-letter condition, respectively, so this factor was pooled. Mean RTs for the conditions are displayed in **Figure 2**.

Main effects were observed for all factors: for Material *F*(1,36) = 9.8, *p* < 0.001, with faster reactions for letters than for non-letter shapes; for Level* F*(1,36) = 58, *p* < 0.001, with faster responses for global than for local level targets and for Congruency* F*(1,36) = 38.1, *p* < 0.001, with faster responses for congruent stimuli than for incongruent stimuli.

There were two-way interactions between Material and Level, *F*(1,36) = 18.2, *p* < 0.001, showing that the difference in response times between global and local targets was larger for non-letter shapes than for letters, as well as between Material and Congruency, *F*(1,36) = 7, *p* = 0.012, showing that the Congruency effect was larger for letters than for non-letter shapes. In addition to this, we observed a three-way interaction between Material, Level and Congruency, *F*(1,36) = 6.7, *p* = 0.014, showing that the Congruency effect differed between letters and non-letter shapes, depending on whether participants were asked to respond to the global or the local level. Due to the three-way interaction, we then analyzed the data separately for letters and for non-letters.

For non-letters, participants responded faster to congruent compared to incongruent targets, *F*(1,36) = 7.4, *p* = 0.01 (congruence effect), and to global compared to local targets, *F*(1,36) = 54.4, *p* < 0.001 (global advantage effect). We obtained an interaction between target level and congruency, *F*(1,36) = 5.5, *p* = 0.025, with greater interference from the global level when participants attended to the local level, *t*(36) = 3, *p* = 0.005, but no interference from the local level to the global, *t* < 1, *p* = 0.9 (asymmetric congruence effect).

For letters, there was a main effect of Congruency, *F*(1,36) = 26.5, *p* < 0.001, with slower responses to incongruent compared to congruent targets (congruence effect). This effect did not differ depending on the level of the target, *F*(1,36) = 2.6, *p* = 0.118, and there was interference both from the local level when attending to the global targets, *t*(36) = 5.1, *p* < 0.001, as from the global level when attending to the local targets, *t*(36) = 2.9, *p* = 0.006.

## DISCUSSION

We investigated the GPE for Navon’s (1977) compound figures, i.e., *global advantage* in combination with an *asymmetric congruence* effect, comparing letters and non-letter shapes, which were expected to differentiate in their GPE. We used a variant of the classical Navon-paradigm, with central presentation and without positional uncertainty, and a specific combination of visual angles for the local and global level of the stimuli.

Central presentation was used, because we were interested in emulating the conditions of reading on the global–local task. In reading, graphemic (phonological), morphological or lexical decoding and identification is limited to what is centrally present during a fixation, typically a word (see Pollatsek, 1993, for an overview), as can be demonstrated in eye-movement studies using gaze- contingent display change techniques (Rayner et al., 1986). Thus, central presentation is a necessary condition for the expected differentiation between letters and non-letters to occur (Plomp et al., 2010). This is consistent with the fact that for peripheral presentation the GPE is robust for non-letters and for letters alike.

For compound figures presented centrally a survey of the literature confirmed that we would be most likely to observe a letter-specific effect, if we chose a stimulus dimensions that are typically encountered in reading. For the local level we imposed a scale of stimuli of about 0.5° of visual angle, close to the critical threshold for fluent reading (Legge et al., 1985; Jordan and Martin, 1987). For the global level, we expected it to fall within the functional visual field (Sanders, 1970). These dimensions are consistent with previous observations on the GPE, which has been reported to disappear under those conditions. However, to our knowledge, we are the first to report for these specific dimensions a comparison between letters and non-letters, the choice of which is motivated by our theoretical assumptions about the role of letter-specific processing as a consequence of automatized reading skills.

In the present study, we obtained under these conditions a differentiation in the GPE between letters and non-letters: The GPE remained intact for non-letter stimuli but disappears for letters; for letters there is no general advantage for global stimuli (no global advantage effect) and the congruence effect is independent of local–global target level (no asymmetric congruence effect). Since the “forest before tree” effect vanished only for letters, we may consider it likely that a letter-specific strategy is applied to these stimuli.

The emergence of a letter-specific strategy is in accordance with earlier studies, in which skilled readers used a specific processing strategy for encoding letters (Lachmann and van Leeuwen, 2004, 2008a; van Leeuwen and Lachmann, 2004), while illiterates did not (Lachmann et al., 2012; see also Fernandes et al., 2014). This letter-specific processing strategy was described as more analytic than for non-letter shapes, for which processing may be called holistic. Consequently, the results suggest that the differentiation of holistic processing for non-letters versus analytic processing of letters can be extended to compound figures, as long as the stimulus dimensions invite a reading-specific strategy.

We do not wish to claim that our conditions closely resemble those of reading. The dimensions of our hierarchical letters are similar to single letters embedded in whole words, but the latter mostly involve different rather than uniform letters, and larger variety at the level of the whole, not to mention lexical, sentence and overall semantic context. Nevertheless, these results may be considered as a small but important step in extending our earlier results to contextually embedded letters.

Comparisons between letter and non-letter stimuli in Navon-local-global settings have rarely been made. For peripheral presentation, Dulaney and Marks (2007) found a GPE for both stimulus categories, as we would expect, since the analytic mode works only with central presentation. Peresotti et al. (1991) presented letter and non-letter stimuli both centrally within a variant of the Navon-local-global design, in order to investigate certain aspects of the time course of information processing (in particular, at what stage the GPE occurs, the perceptual or the decision level, Miller, 1981; Navon, 1981b). To this aim it was sufficient to have “letters vs. non-letters” as a variation only at the global level. Their study, therefore, did not involve a *systematic* comparison of the GPE in letters and non-letter shapes. For central presentation, this has, to our knowledge, only been done in a study by Poirel et al. (2008b) and an EEG follow-up (Beaucousin et al., 2011). Their results seem to contrast with ours. The main distinction these authors obtained was between meaningful (both letters and non-letter objects) and meaningless material (random scribbles). They found that the global level of hierarchical stimuli was always processed faster than the local level (global advantage), irrespective of meaningfulness; however, the *asymmetric congruence* effect (exclusive global-to-local interference), was restricted to meaningful stimuli only. This latter category included both meaningful objects and letters.

However, Poirel et al. (2008b) used a relatively large visual angle: for local items >1° (height) and for global items >11 (width). In the present study, local and global targets were approximately half those respective sizes. In other words, the local level letters are beyond the optimal size for reading (Legge et al., 1985) and thus for the analytic strategy, whereas the global level exceeds the functional visual field (Sanders, 1970; Motter and Simioni, 2008). In this respect, the results of Poirel et al. (2008b) do not contradict to our approach. Parts of their results do not fit, however, with the earlier studies in this field, which found no GPE for letters with the visual angles used by Poirel and his associates (Kinchla and Wolfe, 1979; Lamb and Robertson, 1990; Luna et al., 1995).

A possible reason for this discrepancy in the literature may be that, at least for letters, GPE effects also depend on the task. Poirel et al. (2008b) involved target detection; most tasks in the literature involved target discrimination. The latter may be more likely to elicit analytic processing. In our previous studies we observed task-dependency using a variety of target discrimination tasks. These tasks, however, used flankers (Eriksen and Eriksen, 1974): letters or non-letters were presented in isolation or surrounded with a non-target shape, which could either be similar (congruent) or different (incongruent) in form. Non-letters were classified faster if the target and its surrounding were form-congruent (e.g., a pseudo-A surrounded by a triangle) as compared to when they differed in shape, i.e., when both were form-incongruent (e.g., a pseudo-A surrounded by a square). We reasoned that non-letter shapes are processed in a holistic mode, in which the central target was perceptually bound to its surrounding. For letter targets no such effect was found in normally reading adults (Lachmann and van Leeuwen, 2004, 2008a). Thus, while non-letter processing generally benefits from surrounding flankers if their surrounding shapes are congruent, letters do not (see also Fernandes et al., 2014). This implies that the surroundings were perceived as separate from the letter target.

In the flanker tasks, in some cases an effect even opposite to congruence occurred with letters (van Leeuwen and Lachmann, 2004); letters are categorized faster when surrounded by an incongruent non-target (e.g., An “A” surrounded by a square) than when the non-target was congruent (e.g., An “A” surrounded by a triangle) – a *negative* congruence effect. This effect occurs because the surrounding context undermines the preferred mode of processing and is therefore actively suppressed; this, presumably, is harder when the surrounding is congruent to the target (Briand, 1994; van Leeuwen and Bakker, 1995; Bavelier et al., 2000). In van Leeuwen and Lachmann (2004), letters in *in*congruent surroundings were processed as efficiently as letters in isolation. Therefore the negative congruence effect suggests that congruency can selectively weaken the analytical processing mode; congruent configurations are, by definition, better Gestalts, and their processing as global wholes will therefore be more difficult to suppress. We may call this “overexpression” of the analytical processing mode: it may sometimes occur habitually, even if it is not optimal for the task.

Whereas in Fernandes et al. (2014), the differentiation in flanker effects was found to be underdeveloped in dyslexic children, in Lachmann and van Leeuwen (2007) it was overexpressed in a subgroup of dyslexics. As this illustrates, the symptom does not necessarily equal the underlying cognitive deficit (Frith, 2001). The observed emphasis on analytic processing may well be the result of a coping strategy; perhaps encouraged by their remedial teaching environment. In analogy to the acoustic domain, where deficient phonological awareness may be a symptom of an underlying, in this case, acoustic deficit (Vandermosten et al., 2010; Groth et al., 2011; Steinbrink et al., 2012), there may likewise be an underlying deficit for the visual domain. We suggest that this deficit is manifested in habitual less-than optimal usage of the analytic strategy.

The flanker studies, in which the visual angle was between 2.6 and 3.5 for the targets, and between 5.2 and 8° for their irrelevant surroundings (Lachmann and van Leeuwen, 2004, 2007, 2008a,b; van Leeuwen and Lachmann, 2004; Jincho et al., 2008), offer insight in the question why normal readers would adopt an analytic mode for letter discrimination in reading. In distinguishing letters, component features are important rather than their global shape distinctions. In van Leeuwen and Lachmann (2004) we varied the task in the following way: one version in which for instance, the response alternatives involved a decision on component features (Category 1 = “A” or “circle” versus Category 2 = “C” or “triangle”) versus one in which response alternatives were based on global shape (Category 1 = “A” or “triangle” versus Category 2 = “C” or “circle”). Whereas the former reproduced the negative congruence effect for letters as opposed to a congruence effect for non-letters, congruence effects were obtained for both letters and non-letters in the latter condition. The upshot is that the preference for analytical strategies is functional and independent of the physical stimulus characteristics. It occurs if the task either requires or benefits from such a letter-specific processing mode and, sometimes, manifests itself even when it is not beneficial for the task, since reading has made this mode habitual for letters, such that it cannot always be suppressed (Lachmann and van Leeuwen, 2007). Thus, it is the reading-specific processing mode that makes the perception of letter special, not their configurational properties (e.g., symmetry) as such; neither their omnipresence, nor the fact that we are extensively trained to decode them.

The present results are consistent with our flanker studies, in suggesting that there is a strategic preference for analytic processing in letters, and that this preference may be context-sensitive and at the same time habitual. According to this reasoning, a notable discrepancy might seem to arise: in the flanker studies analytic processing leads to the decrease of congruence effects, or even their reversal; in compound stimuli it results in an increase in congruence effects, as these now occur both ways between the local and global levels. However, this discrepancy might be only apparent: the flanker congruency effects are clearly of perceptual origin (Boenke et al., 2009) and result from spurious feature binding. Whereas event-related potentials studies have found these processes to coincide with the GPE effect around 200 ms (Han et al., 2003), others have shown the GPE to arise earlier, i.e., around 100 ms, and thus to be of sensory origin (Proverbio et al., 1998). We may assume the latter without compromising our assumption that the effects of analytic processing of letters are context-dependent.

Context-dependency of analytic processing is not confined to letter studies only. When the task is to detect a part of a jigsaw puzzle piece that would prevent it to fit with another piece (Hogeboom and van Leeuwen, 1997), as long as the pieces are not too complex the global symmetry of the pieces influences the detectability of the target, meaning that perception is holistic. With increased complexity, the global symmetry is ignored, i.e., perception is analytic, and the parts of the figure are scanned in a serial manner (for a similar distinction, see Roelfsema and Houtkamp, 2011).

We believe it is not stimulus complexity per se that determines strategy. Task difficulty can be another factor. The Indian illiterates in Lachmann et al. (2012) performed the flanker task analytically for both letters and non-letters. They used analytic processing, in spite of having had minimal exposure to Western culture and education, known to promote context-free processing (Ventura et al., 2008b). This may illustrate our claim that analytic processing is a resident skill, not something acquired during training. The illiterates used analytic processing for both letters and non-letters because both are unfamiliar and the task, therefore, is rated to be difficult. This is reflected by very high RT of the illiterates as compared to skilled readers.

Task requirements can be another factor in whether perception is holistic or analytic. We discussed an example (van Leeuwen and Lachmann, 2004) where in the flanker experiment the task requirements invoked a shift from analytic to holistic processing in letters. Clearly, the ability to process letters holistically is not lost as a result of having learned to read (e.g., Borst et al., 2014). Likewise, switching to an analytic processing strategy for non-letters remains possible. With non-letter shapes only, in a part-whole detection task, presenting another part as preceding context can prime a certain configuration. This effect also depends on the task: when for the same figures the task is changed, such that no longer the part-whole structure but only a figural detail is relevant, the perceptual strategy becomes analytic and the preceding context is ignored (Stins and van Leeuwen, 1993). The observation that task requirements led to a shift between holistic and analytic processing may explain why the results by Poirel et al. (2008b) stand aside from the other studies in the literature: compared to their studies the latter may be seen as having a greater emphasis on analytic processing.

## GENERAL CONCLUSION

Reading is a secondary process and its acquisition involves long-lasting and gradual procedural learning (Fawcett, 2002; Lachmann, 2002; Nicolson et al., 2010), during which already established visual and auditory functions are recruited and modified in a way to guarantee fast and accurate decoding of orthographic symbols. This involves the recruitment of processing strategies optimal for reading, and getting these optimally coordinated (Lachmann, 2002). Once functional coordination is optimized, the coordinated skill gets automatized (Fawcett, 2002; Lachmann, 2002). All this takes about 3–4 years (Rayner and Pollatsek, 1989; Lachmann and van Leeuwen, 2008b). As a result, letters are detected and processed automatically in a cross-modal fashion (Blomert, 2011); the specific set of fine-tuned processing strategies is habitual activated whenever it comes to situations of reading or to tasks where letter-specific processing makes sense. As a consequence, information processing in these situations is very fast and still accurate. Suboptimal functional coordination and its subsequent automatization, however, may lead to reading disability (Badian, 2005; Rusiak et al., 2007; Lachmann et al., 2009; Blomert, 2011; Perea et al., 2011; Perea and Panadero, 2014).

The automatization of letter-specific processing while learning to read seems not to result in losing any perceptual skills, but in acquiring habits that sometimes lead to suboptimal performance on certain tasks, for instance ones involving symmetry in letters (Lachmann and van Leeuwen, 2007). If reading involves the build-up of abstract or cross-modal letter codes, from which phonological information can readily be accessed, holistic information can interfere, and is therefore better ignored or, when needed, actively suppressed. For letters, the relevant context is not the level of graphemic representations of other letters, but their cross-modal encodings and the lexical items of which they are part.

## Conflict of Interest Statement

The authors declare that the research was conducted in the absence of any commercial or financial relationships that could be construed as a potential conflict of interest.
